# TracerX: Dynamic Symbolic Execution with Interpolation (Competition Contribution)

**DOI:** 10.1007/978-3-030-45234-6_28

**Published:** 2020-03-13

**Authors:** Joxan Jaffar, Rasool Maghareh, Sangharatna Godboley, Xuan-Linh Ha

**Affiliations:** 8 University of Paderborn, Paderborn, Germany; 9 ICREA, Open University of Catalonia, Barcelona, Spain; National University of Singapore, Singapore, Singapore

**Keywords:** Dynamic Symbolic Execution, Interpolation, Testing, Code Coverage

## Abstract

Dynamic Symbolic Execution (DSE) is an important method for testing of programs. An important system on DSE is KLEE [1] which inputs a C/C++ program annotated with symbolic variables, compiles it into LLVM, and then emulates the execution paths of LLVM using a specified backtracking strategy. The major challenge in symbolic execution is *path explosion*. The method of *abstraction learning* [7] has been used to address this. The key step here is the computation of an *interpolant* to represent the learned abstraction.

TracerX, our tool, is built on top of KLEE and it implements and utilizes *abstraction learning*. The core feature in abstraction learning is *subsumption* of paths whose traversals are deemed to no longer be necessary due to similarity with already-traversed paths. Despite the overhead of computing interpolants, the *pruning* of the symbolic execution tree that interpolants provide often brings significant overall benefits. In particular, TracerX can *fully* explore many programs that would be impossible for any non-pruning system like KLEE to do so.

## 1 Overview and Software Architecture

Symbolic execution has emerged as an important method to reason about programs, in both verification and testing. By reasoning about inputs as symbolic entities, its fundamental advantage over traditional black-box testing, which uses concrete inputs, is simply that it has better *coverage* of *program paths*. In particular, *dynamic symbolic execution* (DSE), where the execution space is explored *path-by-path*, has been shown effective in systems such as DART [4] and KLEE [1]. A key advantage of DSE is that by examining a single path, the analysis can be both precise, and efficient. However, the key disadvantage of DSE is that the number of program paths is in general *exponential* in the program size, and most available implementations of DSE do not employ a general technique to prune away some paths.

In TracerX, our primary objective is to address the path explosion problem in DSE. More specifically, we wish to perform path-by-path exploration of DSE to enjoy its benefits, but we include a *pruning mechanism* so that a generated path can be eliminated if it is guaranteed not to violate the stated safety conditions. Toward this goal, we employ the method of *abstraction learning* [7], which is more popularly known as *lazy annotations* [8,9].

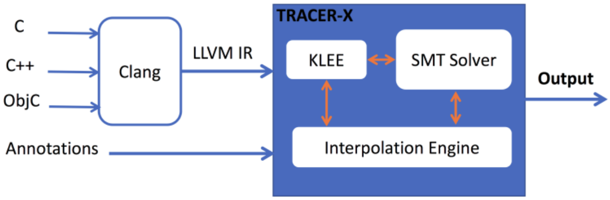

**Fig. 1.** TracerX Framework

The software architecture of TracerX is presented in Fig. 1. The core feature of TracerX is the use of *interpolation*, which serves to generalize the context of a node in the symbolic execution tree (SET) with an approximation of the weakest precondition of the node. This method was implemented in the TRACER system [6], which was the first system to demonstrate DSE with pruning. TRACER was primarily used to evaluate new algorithms in verification, analysis and testing, e.g., [2,3,5]. While TRACER was able to perform bounded verification and testing on many examples, it could not accommodate industrial programs which often dynamically manipulate the heap memory. TracerX combines the state-of-the-art DSE technology used in KLEE with the pruning technology in TRACER to address this issue.

```
x = 0;
if ( b1 ) x += 12;
if ( b2 ) x += 15;
assert (x != 28);
```

**Fig. 2.** A Sample Program

Now we explain *interpolation* in more detail. While exploring the SET, an *interpolant* of a state is an *abstraction* of it which ensures the safety of the subtree rooted at that state. In other words, if we continue the execution with the interpolant instead of the state we will *not* reach any error. Thus, upon encountering another state of the same program point, if the context of the state *implies* the interpolant formula, then continuing the execution from the new state will not lead to any error. Consequently, we can prune the subtree rooted at the new state.

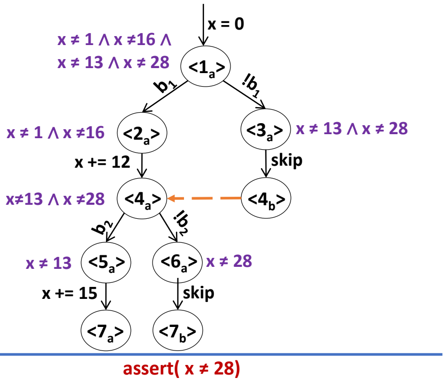

**Fig. 3.** SET with Interpolation of Program in Fig. 2

*Example 1.* Consider the program in Fig. 2 and its SET explored by SE with interpolation in Fig. 3. The variables b1, b2 are symbolic and all combinations of the boolean conditions are satisfiable. The final statement assert$(x \neq 28)$ is the target. The path condition for every path is shown in the set in black color.

We traverse the SET in a left-right depth-first manner. In the end of the first path $x = 27$ which does not violate the assertion. Considering the target and the update on variable $x$ between $\langle 5_a \rangle$ and $\langle 7_a \rangle$, we generate an interpolant which store the weakest precondition at $\langle 5_a \rangle$: $x \neq 13$ (Shown in purple color). Similarly, an interpolant is also computed at $\langle 6_a \rangle$: $x \neq 28$.

Now, combining these two interpolants, we generate an interpolant for the node $\langle 4_a \rangle$. Note that the weakest precondition here is $b_2 \longrightarrow (x \neq 13) \wedge !b_2 \longrightarrow (x \neq 28)$. We approximate this formula with the conjunction $(x \neq 13) \wedge (x \neq 28)$. Next, moving to $\langle 2_a \rangle$, the interpolant at $\langle 4_a \rangle$ is received and considering the update on variable $x$ between $\langle 2_a \rangle$ and $\langle 4_a \rangle$, an interpolant is generated at $\langle 2_a \rangle$: $x \neq 1 \ \wedge \ x \neq 16$. Now moving to $\langle 4_b \rangle$, we check if the path condition at $\langle 4_b \rangle$ $(x = 0 \wedge !b_1 \wedge \mathit{skip})$ implies the interpolant that was generated at $\langle 4_a \rangle$ $(x \neq 13 \ \wedge \ x \neq 28)$. Since the implication holds, node $\langle 4_b \rangle$ is subsumed with node $\langle 4_a \rangle$ (indicated by orange arrow) and the subtree below $\langle 4_b \rangle$ is pruned. The SET traversal continues by computing the interpolant at $\langle 3_a \rangle$ which is computed from $x \neq 13 \ \wedge \ x \neq 28$ subsuming $\langle 4_b \rangle$ and the updates between $\langle 3_a \rangle$ and $\langle 4_b \rangle$ (which is `skip`). The interpolants at $\langle 2_a \rangle$ and $\langle 3_a \rangle$ are then combined to generate an interpolant at $\langle 1_a \rangle$: $x \neq 1 \ \wedge \ x \neq 16 \ \wedge \ x \neq 13 \ \wedge \ x \neq 28$. Note that KLEE would explore the 4 paths in the SET while TracerX explores only two paths to the end. □

## 2 Discussion on Strengths and Weaknesses

In Test-Comp 2020, TracerX stood at 6th rank in overall. Inspecting the results, TracerX was one of the teams having the highest score in: `cover-branches.BitVectors` and `cover-error.ControlFlow`. Moreover, TracerX was one of the top 3 scorers in: `cover-branches.DeviceDriversLinux64`, `cover-branches.ControlFlow`, and `cover-error.BitVectors`.

TracerX also accomplished more tasks by a meaningful margin compared to KLEE in: `cover-branches.BusyBox` and `cover-branches.MainHeap`. On the other hand, TracerX performed poorly in 3 sub-categories: `cover-error.ReachSafety-ECA`, `ReachSafety-Sequentialized` (both branches) and `cover-error.Floats`[1].

We should emphasize that TracerX in general requires symbolic execution trees to be bounded. Otherwise, interpolants cannot be computed. Moreover, TracerX is a heavy-weight approach and the overhead pays off as the problems gets harder. As a result it is expected for other light-weight approaches to have better results compared to TracerX in short timeout and memory limits.

Moreover, it appears that the configuration we used to explore unbounded programs (max-depth=1000) and also in the *benchexec tool-info* (wrongly running TracerX with the default memory (2GB) instead of 15GB RAM) might have had a profound effect in reaching timeout on the test programs.

[1] TracerX does not support symbolic expressions over floating point arithmetic.

## 3 Tool Setup and Configuration

The TracerX version used in TEST-COMP 2020 is available at https://gitlab.com/sosy-lab/test-comp/archives-2020/blob/testcomp20/2020/tracerx.zip[2]. The configuration/setting and running of TracerX is similar to KLEE. TracerX has some extra command line arguments. Firstly, the argument "solver-backend=z3" should be provided to run TracerX with interpolation. Without this option TracerX will run similar to KLEE. TracerX can do exploration in both the Random and DFS modes. However, the DFS exploration mode (using "-search=dfs") is preferred since it naturally increases the chance of generating interpolants. Furthermore, the option "-subsumed-test" should be used to generate a test-case from the subsumed nodes. This option is required for the coverage competition. The following is a sample full command line after compiling and running `tracerx.py`:

"`../tracerx-svcomp/bin/../tracerx_build/Release+Asserts/bin/klee -max-memory=14305 -output-dir=../tracerx-svcomp/bin/../test-suite -search=dfs -solver-backend=z3 -write-xml-tests -tc-orig=s3_clnt_3.BV.c.cil-2a.c -tc-hash=acd2272114f13977ea7bdc712c7567ec2e43dc8e07ef033eb67487bab7f66d59 --dump-states-on-halt=false -exit-on-error-type=Assert -max-depth=1000 -max-time=900 /tmp/tmpvwkb459r/s3_clnt_3.BV.c.cil-2a.c.bc`"

The two command line options, "-max-memory" and "-max-time" are used to set the maximum memory and time budget. The options "-write-xml-tests", "-tc-orig", and "-tc-hash" are to record the test input information. Once the halt instruction is invoked, "-dump-states-on-halt" creates a test case from all active states[3]. The option "-exit-on-error-type=Assert" terminates the search as soon as a bug is found (used only for coverage categories). The command line option "-max-depth=1000" is used to bound the maximum number of branches explored in unbounded paths.

## 4 Software Project and Contributors

The information about TracerX with self-contained binary is publicly available at https://www.comp.nus.edu.sg/~tracerx/. Also, the source code can be accessed at https://github.com/tracer-x/klee repository. Authors of this paper and other colleagues have contributed and developed TracerX at National University of Singapore, Singapore. The authors of this paper acknowledge the direct and indirect support of their students, former researchers, and colleagues.

[2] The *benchexec* tool-info file is https://github.com/sosy-lab/benchexec/blob/master/benchexec/tools/tracerx.py and the benchmark description file is https://gitlab.com/sosy-lab/test-comp/bench-defs/blob/master/benchmark-defs/tracerx.xml.

[3] This was disabled to save execution time. However, it would have been better to enable this option for maximum coverage.
